# KCNQ1 Polymorphism in the Context of Ischemic Cardiomyopathy: A Potential Key to Decision‐Making for Device Implantation

**DOI:** 10.1002/clc.70148

**Published:** 2025-05-14

**Authors:** Uğur Özkan, Metin Budak, Muhammet Gürdoğan, Gülnur Öztürk, Mustafa Yildiz, Gökay Taylan, Servet Altay, Kenan Yalta

**Affiliations:** ^1^ Department of Cardiology, School of Medicine Trakya University Türkiye; ^2^ Department of Biophysics, Faculty of Medicine Trakya University Türkiye; ^3^ Department of Physiotherapy and Rehabilitation, Faculty of Health Sciences Trakya University Türkiye

**Keywords:** implantable cardioverter‐defibrillator, ischemic cardiomyopathy, KCNQ1 gene polymorphism, ventricular tachyarrhythmia

## Abstract

**Background:**

Ventricular tachyarrhythmia (VTA) in ischemic cardiomyopathy (ICM) is a life‐threatening condition influenced by genetic factors and electrical remodeling. This study investigated the association between KCNQ1 gene polymorphisms (rs2237892 and rs2237895) and the development of VTA in ICM patients to improve risk stratification and guide device implantation decisions.

**Methods:**

This single‐center study included 213 ICM patients with implantable cardioverter‐defibrillators (ICD) for primary prevention of VTA. Patients were divided into arrhythmia and control groups based on device interrogation findings. Genetic analysis for rs2237892 and rs2237895 polymorphisms was performed using real‐time polymerase chain reaction (PCR). Clinical, electrocardiographic, and laboratory parameters were analyzed. Correlation and logistic regression analyses evaluated the association between KCNQ1 polymorphisms and VTA risk.

**Results:**

The arrhythmia group demonstrated significantly higher QT dispersion, frontal QRS‐T angle, and T‐wave peak‐to‐end interval compared to the control group. The TT genotype of rs2237892 and the AC genotype of rs2237895 were significantly associated with increased VTA risk (*p* < 0.001). Multivariate analysis confirmed these genotypes as independent predictors of VTA. No significant differences in other clinical or laboratory risk factors were observed.

**Conclusions:**

KCNQ1 gene polymorphisms (rs2237892 and rs2237895) are strongly associated with VTA in ICM patients, suggesting a potential role as biomarkers for risk stratification. These findings may assist in tailoring ICD implantation decisions and improving patient outcomes.

## Graphical abstract

Potassium Channels and Ventricular Arrhythmia Risk. Altered potassium channel function modifies cardiac repolarization: gain‐of‐function shortens, loss‐of‐function prolongs the QT interval. Both increase susceptibility to ventricular tachyarrhythmias, especially with myocardial scarring. Shown: action potential phases, ionic currents, and key channel contributions.

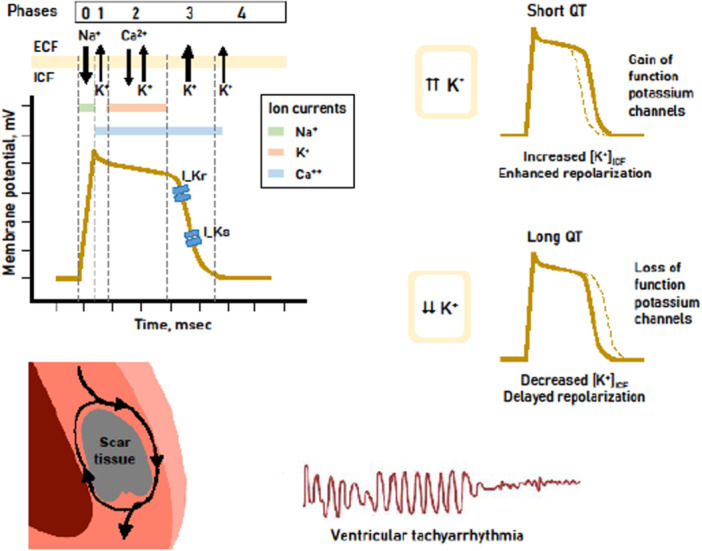

## 1 Introduction

Ventricular tachyarrhythmia (VTA) is a clinical condition characterized by abnormal electrical activation in the myocardial tissue, often accompanied by hemodynamic collapse, and is associated with high morbidity and mortality rates [1]. VTA arises primarily from two main substrates: fibrous tissue, which commonly develops in the context of ischemic heart disease, and/or ongoing chronic ischemia. Scar tissue is involved as the nidus in the most common mechanism for the development of tachyarrhythmia featuring the re‐entry circuit. The excitation wave can re‐enter and create a loop around the nidus, facilitated by slow conduction or an electrical block [2]. Ischemia induces electrical remodeling that triggers arrhythmia by affecting ion channel function and myocyte activity [3]. Mechanisms affecting the VTA can be observed across a broad clinical spectrum, ranging from myocardial damage and inflammation associated with acute coronary syndrome to stable ischemic heart disease. Advances in the diagnosis and treatment of cardiovascular diseases have significantly reduced the incidence of adverse cardiac events, including premature death. However, this improvement in early survival rates has increased the likelihood and frequency of complications, such as ischemic cardiomyopathy, arrhythmias, and recurrent ischemia over time [4]. Then again, these complications, which are associated with high mortality and morbidity rates, do not develop in every patient who serves as a substrate.

Potassium Voltage‐Gated Channel Subfamily Q Member 1 (KCNQ1) belongs to a gene family that encodes potassium channels. KCNQ1 encodes the K_v_7.1 (KvLQT1) protein, which regulates the levels of potassium ions that affect repolarization, action potential duration (APD), excitability, and rhythm in myocytes [5]. It has been demonstrated in the literature that mutations in this gene can lead to arrhythmic conditions such as long QT Syndrome (LQT1) and the Jervell and Lange‐Nielsen syndrome (J‐LN) [6, 7]. However, the impact of KCNQ1 polymorphisms on the development of ventricular arrhythmias in patients with ischemic cardiomyopathy has not yet been investigated.

In view of the foregoing, we carried out this study to investigate whether there is a relationship between KCNQ1 polymorphisms and the development of ventricular arrhythmias in a group of patients with ischemic cardiomyopathy who are predisposed to ventricular arrhythmias.

## 2 Materials and Methods

### 2.1 Population and Sample

The population of this single‐center study consisted of patients with a history of ischemic cardiomyopathy who underwent intracardiac defibrillator (ICD) implantation between January 1st, 2017, and July 1st, 2024, based on established guidelines [8, 9]. Patients with a diagnosis of ventricular arrhythmia were identified using a device programmer and evaluated by two cardiologists [10]. The absence of ongoing ischemic foci that could trigger VTA development in patients was confirmed using diagnostic coronary angiography and/or noninvasive ischemic tests, i.e., myocardial perfusion scintigraphy and/or coronary computed tomography.

Patients with coronary ischemia that could cause VTA, those with an ejection fraction (EF) greater than 35%, those who underwent device implantation due to secondary causes that could lead to arrhythmia, such as arrhythmogenic right ventricular cardiomyopathy, long QT syndrome, Brugada syndrome, hypertrophic cardiomyopathy, and congenital structural heart disease, those with nonischemic cardiomyopathy requiring device implantation, those with chronic autoinflammatory diseases, those who have been using Class III antiarrhythmic medications for reasons other than ventricular tachyarrhythmia, e.g., rhythm control for atrial fibrillation (AF), those with prolonged QT intervals on electrocardiogram (ECG) secondary to medications, including antiarrhythmics, antipsychotics and antibiotics. In the end, the arrhythmia group consisted of 213 patients diagnosed with ischemic cardiomyopathy who underwent ICD implantation and volunteered to participate in the study, whereas the control group consisted of patients without arrhythmias. Participants included in the arrhythmia and control groups were selected from among patients who attended routine follow‐up visits to the cardiac outpatient clinic or presented to the emergency department due to device shock.

The study protocol was approved by the Trakya University Medical Faculty Ethics Committee (TUTF‐BAEK 2022/105). The study was conducted in accordance with the ethical considerations outlined in the Declaration of Helsinki.

### 2.2 Data Collection

The participants’ sociodemographic characteristics, medical histories, and the medications they have been using were obtained from the anamneses taken during the examination. The participants’ laboratory test results and echocardiographic and angiographic data were obtained from the hospital automation system. All metabolic panel tests were conducted using an autoanalyzer (Roche Diagnostic Modular Systems, Tokyo, Japan). Blood samples (5 cc) were collected from participants in both groups into tubes containing ethylenediaminetetraacetic acid (EDTA). Two genotypes of the KCNQ1 gene were identified using fluorescein‐labeled real‐time polymerase chain reaction (PCR) (Applied Biosystems 7500 Fast Instrument) with a fluorescent marker (Taqman) after isolating the deoxyribonucleic acid (DNA) from the blood samples.

### 2.3 Genotyping

DNA isolation from EDTA‐treated whole blood was performed according to the manufacturer's instructions (catalog number 761001D; Thermo Fisher Scientific, USA). Real‐time PCR assays were performed for rs2237892 and rs2237895 polymorphisms with TaqMan single‐nucleotide polymorphism (SNP) probes using 2 µL DNA samples from each participant (catalog number 4351379 for rs2237892 and 4351379 for rs2237895, Thermo Fisher Scientific, USA). All PCR assays were performed in real time using the Applied Biosystems 7500 Fast instrument with initial denaturation at 94°C for 5 min, subsequently at 94°C, 57°C and 72°C for 30 s each, and finally at 72°C for 10 min. Genotyping and melt curve analyses were conducted at the end of each cycle [11].

#### 2.3.1 Ventricular Tachyarrhythmia Diagnosis

The diagnosis of VTA was established using a device programmer in patients with implanted devices equipped with ICD. Rhythm analysis featured parameters such as cycle length, i.e., the interval in milliseconds between two sensed beats by the ICD, rate branch, i.e., comparison of ventricular and atrial rates, interval stability, i.e., whether the intervals are consistent, onset type, i.e., whether sudden or gradual, and morphology discrimination [8, 10, 12].

#### 2.3.2 ECG Analysis

QT interval was defined as the longest duration from the beginning of the Q wave to the end of the T wave on ECG [13]. The corrected QT (QT_c_) duration was calculated using the Bazett's formula [14]. The T‐wave peak‐to‐end interval (TPEI) was measured from all leads using the “Tangent Method” [13]. The frontal QRS‐T angle was calculated as the difference between the QRS axis (ventricular depolarization) and the T‐wave axis (ventricular repolarization) on a standard 12‐lead ECG [15].

### 2.4 Statistical Analysis

SPSS 26.0 (Statistical Product and Service Solutions for Windows, Version 26.0, IBM Corp., Armonk, NY, U.S., 2019) software package was used to conduct the statistical analyses of the collected data. The results of the statistical analyses were expressed using descriptive statistics, i.e., mean ± standard deviation values in the case of continuous variables determined to conform to the normal distribution, median with minimum and maximum values in the case of continuous variables determined to not conform to the normal distribution, and numbers and percentage values in the case of categorical variables. The normal distribution characteristics of numerical variables were analyzed using the Shapiro‐Wilk test. In comparing the differences in numerical variables between two independent groups, independent samples t‐test was used for numerical variables determined to conform to the normal distribution, and Mann‐Whitney U test was used for numerical variables determined to not conform to the normal distribution. Additionally, the chi‐squared test was used to compare the differences in categorical variables between the groups. The effects of the variables examined within the scope of the study on the development of VTA were evaluated using univariate analysis. Variables that were determined to have a significant impact on VTA development in univariate analysis were further examined using multivariate analysis. Binary logistic regression analysis was used for both the univariate and multivariate analyses. Correlation analyses were performed to investigate the relationship, if any, between KCNQ1 polymorphisms and indicators of ventricular depolarization and repolarization. Probability (*p*) statistics of ≤ 0.05 were deemed to indicate statistical significance.

## 3 Results

The mean age of the sample, 136 of whom (63.8%) were male, was 66 years. The mean duration of follow‐up period after ICD implantation was 42 months. Patients’ demographic characteristics, laboratory test results, echocardiographic data, and the medications they have been using are given in Table 1. The time elapsed since implantation was significantly shorter in the arrhythmia group compared to the control group, whereas the incidence of VTA was significantly higher (*p* = 0.003). Additionally, inflammatory index markers were elevated in the arrhythmia group compared to the control group. There was no significant difference between the groups in other parameters (*p* > 0.05). The distribution of ECG characteristics and KCNQ1 gene polymorphism results by the study groups is shown in Table 2. The QT interval, QT_c_ interval, QT dispersion (QT_d_), frontal QRS‐T angle, TPEI, and QRS fragmentation were all higher in the arrhythmia group than in the control group.

**Table 1:** Demographic characteristics and labaratory findings of the study populations.

Characteristics	Aritmic (*n* = 62)	Non Aritmic (*n* = 151)	*p*
Male gender	43 (69.4)	93 (61.6)	0.28
Age	66.4 ± 8.2	66.5 ± 7.6	0.92
HT	50 (80.6)	118 (78.1)	0.82
Drinking	5 (8.1)	19 (12.6)	0.34
Smoking	11 (17.7)	14 (9.3)	0.13
Prior Stroke	5 (8.1)	15 (9.9)	0.67
DM	19 (30.6)	40 (26.5)	0.54
Dialysis	5 (8.1)	5 (3.3)	0.13
EF	31 (25–35)	30 (20–35)	0.47
LVD	60.3 ± 3.2	61.2 ± 3.5	0.07
Coronary reperfusion type			
PCI	34a (54.8)	64a (42.4)	0.16
CABG	20a (32.3)	62a (41.1)	
CABG+ valve surgery	7a (11.3)	14a (9.3)	
Hybrid	1a (1.6)	11a (7.3)	
Implantation duration	39.6 ± 8.4	43.5 ± 9.4	0.003
Device type			
Single ICD	48a (77.4)	123a (81.5)	0.76
Dual ICD	2a (3.2)	5a (3.3)	
CRT‐ICD	12a (19.4)	23a (15.2)	
WBC	7.3 ± 0.6	7.3 ± 1	0.8
Lym	2.2 ± 0.8	2.4 ± 0.7	0.051
Neu	5.6 ± 1.1	4.9 ± 0.8	0.001
PLT	213 (88–274)	194 (137–245)	< 0.001
Sodium	140 (136–145)	141 (137–146)	0.57
Potassium	4.6 ± 0.8	4.7 ± 0.6	0.3
Magnesium	2.4 (1.9–2.8)	2.4 (1.8–2.9)	0.43
Creatinin	1.3 (0.6–5.2)	1.3 (0.5–5)	0.39
Hgb	11.3 ± 2	11.7 ± 1.9	0.26
SII	525.5 (165.2–2855.5)	379 (162–1914.4)	< 0.001
Beta blocker	58 (93.5)	139 (92.1)	0.7
SGLT2	10 (16.1)	32 (21.2)	0.39
RAS blocker			
Not using	6 (9.7)	10 (6.6)	0.21
ACEi/ARB	48 (77.4)	131 (86.8)	
Sacubitril valsartan	8 (12.9)	10 (6.6)	
MRA	18 (29)	32 (21.3)	0.3
Loop diuretics	29 (46.8)	63 (42)	0.52
Ranelozin	19 (30.6)	54 (35.8)	0.47
Statin	60 (96.8)	145 (96)	0.79
Hospitalization (month/year)			
No hospitalization	9a (14.5)	16a (10.6)	0.07
1–3	18a (29)	71b (47)	
4–6	13a (21)	26a (17.2)	
7–9	19a (30.6)	29a (19.2)	
9–12	2a (3.2)	9a (6)	
> 12	1a (1.6)	0a (0)	

Abbreviations: ACEi, angiotensin‐converting enzyme inhibitors; ARB, angiotensin receptor blocker; CABG, cardiac bypass greft; DM, diabetes mellitus; EF, ejection fraction; Hgb, hemoglobin; HT, hypertension; ICD, Implantable cardioverter‐defibrillator; LVD, left venticular end‐diastolic diameter; Lym, lymphocyte; MRA, mineralocorticoid receptor antagonist; Neu, neutrophil; PCI, percutaneous coronary ıntervention; PLT, platelet; SII, systemic immune‐inflammation index; WBC, white blood count.

**Table 2:** ECG Findings and KCNQ1 gene polymorphism results of study populations.

Characteristics	Aritmic group	Non‐Aritmic group	
QRS duration	113 (73–450)	121 (90–174)	0.45
QT	391 ± 15.6	384 ± 19.1	0.008
Corrected QT	422.2 ± 20	413.3 ± 19.8	0.004
QT dispersion	90.8 ± 26.7	73.3 ± 24.1	< 0.001
Frontal QRS‐T angle	71.9 ± 35.8	57 ± 23.9	0.003
T wave peak‐to‐end interval	88.8 ± 16.4	73.3 ± 14.1	< 0.001
QRS fragmentation			
No fragmentation	20a (32.3)	83b (55)	0.039
LBBB	9a (14.5)	18a (11.9)	
RBBB	9a (14.5)	18a (11.9)	
Pace ritm	3a (4.8)	5a (3.3)	
Inferior fragmentation	8a (12.9)	5b (3.3)	
Anterior fragmentation	7a (11.3)	13a (8.6)	
Lateral fragmentation	6a (9.7)	9a (6)	
RS2237892 C/T			
CC	57a (91.9)	126a (83.4)	< 0.001
CT	0a (0)	25b (16.6)	
TT	5a (8.1)	0b (0)	
RS2237895 A/C			
AA	10a (16.1)	48b (31.8)	< 0.001
AC	48a (77.4)	71b (47)	
CC	4a (6.5)	32b (21,2)	

Abbreviations: LBBB, left bundle branch block; RBBB, right bundle branch block.

We investigated two SNPs for their effects on the development of VTA. The frequencies of minor allele homozygotes and heterozygotes for the SNPs are given in Table 2. The CT genotype of the RS2237892 polymorphism was not observed in the arrhythmia group, whereas the TT genotype was not observed in the control group (*p* < 0.001 for both cases). There was no significant difference between the groups in the frequency of CC genotype (*p* > 0.05). The AC genotype of the RS2237895 polymorphism was significantly more frequent in the arrhythmia group, whereas the AA and CC genotypes were significantly more frequent in the control group (*p* < 0.001 for both cases).

The results of correlation analysis on the effect of KCNQ1 polymorphism on baseline resting ECG depolarization and repolarization are given in Table 3. The prognostic values of clinical factors, genetic factors, and baseline ECG parameters on the development of VTA were assessed using logistic regression analyses (Tables 4 and 5).

**Table 3:** Correlation Analysis of RS2237892 and RS2237895 Gene Polymorphisms with the Alteration of ECG Parameters in Arrhythmia.

	Aritmia development		Corrected QT		QT dispertion		Frontal QRS‐T angle		T wave peak‐to‐end interval	
	r	*p*	r	*p*	r	*p*	r	*p*	r	*p*
Corrected QT	0.191^b^	0.005								
QT dispersion	0.285^b^	< 0.001	−0.026	0.711						
Frontal QRS‐T angle	0.196^b^	0.004	0.002	0.983	0.572^b^	< 0.001				
T wave peak‐to‐end interval	0.403^b^	< 0.001	0.037	0.590	0.530^b^	< 0.001	0.350^b^	< 0.001		
RS2237892 CC	0.111	0.107	−0.135^a^	0.049	−0.001	0.991	−0.013	0.846	0.088	0.201
RS2237892 CT	−0.234^b^	0.001	0.096	0.165	−0.121	0.078	−0.104	0.129	−0.183^b^	0.007
RS2237892 TT	0.242^b^	< 0.001	0.107	0.121	0.259^b^	< 0.001	0.252^b^	< 0.001	0.188^b^	0.006
RS2237895 AA	−0.160^a^	0.020	−0.014	0.843	−0.689^b^	< 0.001	−0.369^b^	< 0.001	−0.336^b^	< 0.001
RS2237895 AC	0.278^b^	< 0.001	−0.045	0.514	0.846^b^	< 0.001	0.528^b^	< 0.001	0.488^b^	< 0.001
RS2237895 CC	−0.179^b^	0.009	0.076	0.271	−0.303^b^	< 0.001	−0.261^b^	< 0.001	−0.248^b^	< 0.001

a Correlation is significant at the 0.05 level (2‐tailed).

b Correlation is significant at the 0.01 level (2‐tailed).

**Table 4:** Univariate and Multivariate Clinic and ECG Predictors of Ventricular Arrhythmia Development in Patients with Ischemic Cardiomyopathy.

	Univariate analiz		Multivariate analiz	
	Odds Ratio (95% CI)	*p*	Odds Ratio (95% CI)	*p*
Age	0.995 (0.958–1.034)	0.80	1.018 (0.969–1.068)	0.48
Gender	1.411 (0.75–2.655)	0.28	1.578 (0.708–3.516)	0.26
Hypertension	1.165 (0.557–2.44)	0.68	1.552 (0.599–4.024)	0.37
Drinking	0.609 (0.217–1.712)	0.35	0.585 (0.147–2.329)	0.45
Smoking	2.111 (0.9–4.951)	0.086	2.982 (1.017–8.744)	0.047
Prior Stroke	0.795 (0.276–2.292)	0.67	1.124 (0.276–4.576)	0.87
Diabetes mellitus	1.226 (0.64–2.348)	0.54	1.136 (0.384–3.361)	0.82
Dialysis	2.561 (0.714–9.183)	0.15	2.54 (0.536–12.034)	0.24
Beta blocker	1.252 (0.388–4.043)	0.71	1.691 (0.339–8.433)	0.52
RAS blocker	1.224 (0.583–2.57)	0.59	1.157 (0.464–2.881)	0.76
SGLT2	0.715 (0.327–1.562)	0.40	0.546 (0.158–1.886)	0.34
MRA	1.509 (0.769–2.958)	0.23	2.275 (0.937–5.522)	0.069
Ranelozin	0.794 (0.421–1.497)	0.48	0.427 (0.182–1.002)	0.051
QRS fragmentation	1.218 (1.059–1.402)	0.006	1.187 (0.984–1.432)	0.074
QRS duration	1.002 (0.993–1.011)	0.71	1.002 (0.992–1.012)	0.71
QT duration	1.021 (1.004–1.038)	0.015	1.021 (0.999–1.044)	0.06
Corrected QT duration	1.022 (1.007–1.038)	0.004	1.023 (1.003–1.043)	0.023
QT dispersion	1.027 (1.015–1.04)	< 0.001	1.009 (0.988–1.031)	0.38
Frontal QRS‐T angle	1.019 (1.008–1.03)	0.001	0.998 (0.981–1.016)	0.84
T wave peak‐to‐end interval	1.073 (1.047–1.1)	< 0.001	1.072 (1.037–1.108)	< 0.001

Abbreviations: MRA, Mineralocorticoid Receptor Antagonist; ns, nonsignificant; SGLT,2: sodium glucose co‐transporter‐2 (SGLT2).

**Table 5:** Univariate and Multivariate Clinic and Genetic Predictors of Ventricular Arrhythmia Development in Patients with Ischemic Cardiomyopathy.

	Univariate analiz		Multivariate analiz	
	Odds Ratio (95% CI)	*p*	Odds Ratio (95% CI)	*p*
Age	0.995 (0.958–1.034)	0.80	1.004 (0.957–1.052)	0.88
Gender	1.411 (0.75–2.655)	0.28	1.288 (0.606–2.737)	0.51
Hypertension	1.165 (0.557–2.44)	0.68	1.589 (0.617–4.091)	0.34
Drinking	0.609 (0.217–1.712)	0.35	0.402 (0.113–1.435)	0.16
Smoking	2.111 (0.9–4.951)	0.09	2.744 (0.931–8.084)	0.067
Prior Stroke	0.795 (0.276–2.292)	0.67	1.15 (0.307–4.312)	0.84
Diabetes mellitus	1.226 (0.64–2.348)	0.54	1.492 (0.559–3.986)	0.43
Dialysis	2.561 (0.714–9.183)	0.15	3.205 (0.667–15.403)	0.15
Beta blocker	1.252 (0.388–4.043)	0.71	0.916 (0.233–3.612)	0.90
RAS blocker	1.224 (0.583–2.57)	0.59	1.339 (0.571–3.14)	0.50
SGLT2	0.715 (0.327–1.562)	0.40	0.385 (0.12–1.242)	0.11
MRA	1.509 (0.769–2.958)	0.23	2.33 (1.008–5.383)	0.048
Ranelozin	0.794 (0.421–1.497)	0.48	0.617 (0.29–1.312)	0.21
RS2237892				
CC	2.262 (0.824–6.209)	0.11	—	ns
CT	—	ns	—	ns
TT	—	ns	—	ns
RS2237895				
AA	0.413 (0.193–0.881)	0.022	2.017 (0.538–7.566)	0.30
AC	3.863 (1.966–7.593)	< 0.001	8.067 (2.453–26.527)	0.001
CC	0.256 (0.087–0.76)	0.014	—	ns

Abbreviations: MRA, Mineralocorticoid Receptor Antagonist; ns, nonsignificant; SGLT‐2, sodium glucose co‐transporter‐2 (SGLT2).

## 4 Discussion

The most notable finding of our study is that the KCNQ1 gene polymorphism independently predicts the development of VTA in patients with ischemic cardiomyopathy. KCNQ1 gene polymorphisms have been studied in association with various clinical conditions. For instance, in patients with long QT syndrome, the minor alleles rs12296050 and rs757092 have been linked to an increased risk of arrhythmias, while the rs2074238‐T allele has been associated with a risk‐reducing or protective effect [16]. Similarly, the rs2237892 and rs2237895 polymorphisms of the KCNQ1 gene have been frequently studied in relation to type 2 diabetes mellitus (T2DM) risk and have also been linked to gestational diabetes [17]. Although previous studies have found KCNQ1 gene polymorphisms to be associated with arrhythmogenic syndromes (such as long QT syndrome, short QT syndrome and familial atrial fibrillation), their effects on arrhythmia development in individuals prone to VTA due to ischemic cardiomyopathy have not been investigated before [18, 19, 20]. Our study provides a novel perspective by investigating KCNQ1 gene polymorphisms in relation to ventricular tachyarrhythmia (VTA) development in patients with ischemic cardiomyopathy for the first time. These polymorphisms were studied in relation with ventricular tachyarrhythmia for the first time and a statistically strong association was found. To the best of our knowledge, our study is the first to demonstrate this relationship.

A decrease in the left ventricular ejection fraction (LVEF) due to loss of myocardial viability is a significant risk factor for sudden cardiac death [2]. This loss of viability occurs particularly in the context of coronary artery disease, which increases the susceptibility to VTA. Multicenter Unsustained Tachycardia Trial (MUSTT) and Multicenter Automatic Defibrillator Implantation Trial II (MADIT‐II) studies have shown that the sudden cardiac death risk occurs when EF falls below 40%, and becomes more pronounced when EF falls below 30% [21, 22]. MUSTT and MADIT‐II studies have indicated that the risk begins with an EF < 40% and becomes more pronounced at an EF < 30% [19, 20]. Solomon et al. reported that this risk is approximately 1.4% per month (< 40% EF) and 2.3% per month (< 30% EF) [23].

Solomon et al. reported the sudden cardiac death risk to be approximately 1.4% (range: 1.2%‐1.6%) per month for EF < 40% and 2.3% (range: 1.8%‐2.8%) per month for EF < 30% [21]. They also noted that this risk began to decrease after the first year, dropping to 0.14% per month after the second year [23]. In such cases, where the incidence of VTA is high, electrophysiological guidelines recommend ICD implantation for prophylaxis [8].

The mean duration of follow‐up of the patients in our sample was 42 months. Arrhythmia developed in 29.1% of the patients during the follow‐up period, consistent with literature data.

The cycles of depolarization and repolarization in ventricular myocytes are largely dependent on the relative changes in the concentrations of potassium, sodium, and calcium ions within the cells, which contribute to the generation of action potentials across the cell membrane. This delicate ionic balance is crucial for determining the membrane potential and the refractory period of myocytes. For ventricular myocytes to be re‐excited after depolarization, the cell must exit the absolute refractory period, begin to stabilize towards the repolarization phase (relative refractory period), and return to the resting membrane potential. In this action potential cycle, sodium and calcium channels primarily govern depolarization, whereas potassium channels are play a key role during the repolarization phase. Due to the critical role of potassium in the action potential, changes in intracellular potassium levels can lead to heterogeneity in ventricular repolarization duration [24, 25]. This heterogeneity significantly contributes to the development of VTA [26, 27].

**Figure 1:**
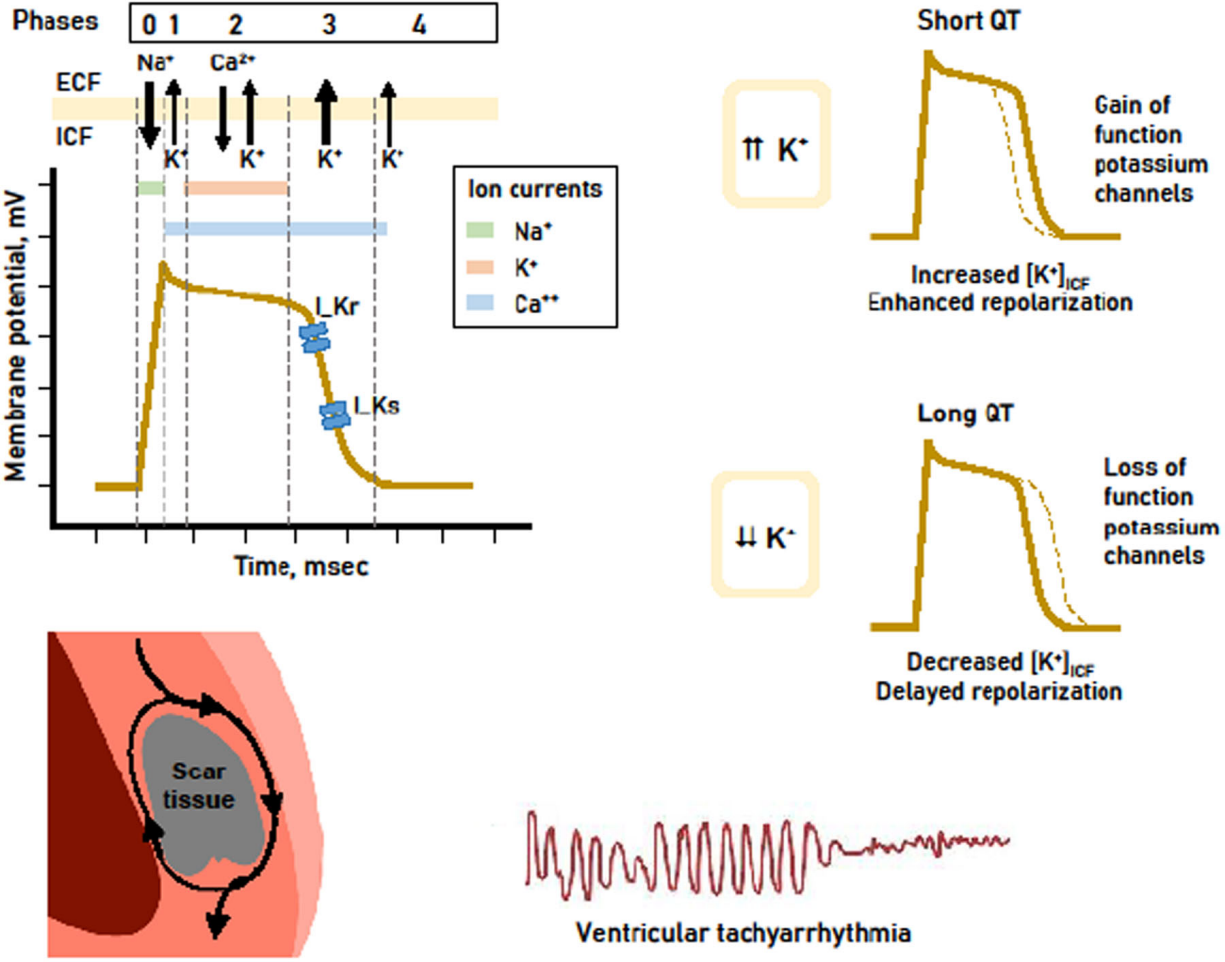
The role of potassium channels in cardiac repolarization: Effects of altered QT duration on ventricular tachyarrhythmias. Schematic representation of cardiac action potential phases and their relationship with ionic currents and potassium channel functions. Gain‐of‐function mutations in potassium channels accelerate repolarization, leading to a shortened QT interval, while loss‐of‐function mutations delay repolarization, prolonging the QT interval. Both scenarios can predispose to ventricular tachyarrhythmias, particularly in the presence of scar tissue. Abbreverations: Ca2 + , Calcium ion; ECF, Extracellular fluid; ICF, Intracellular fluid; I_Kr, Rapid delayed rectifier potassium current; I_Ks, Slow delayed rectifier potassium current; K + , Potassium ion; Na + , Sodium ion.

KCNQ1 encodes a potassium channel that plays a vital role in cardiac repolarization through the generation of a slowly activated delayed rectifier potassium current (I_Ks) [5]. The activation state of this channel is crucial for determining APD. Loss‐of‐function mutations that reduce I_Ks can prolong the repolarization phase, leading to an extension of the APD. For example, the V205M mutation causes a depolarization shift in the activation voltage and accelerates channel deactivation, resulting in a reduction in I_Ks. This extension of APD due to altered repolarization results in a prolonged QT interval (long QT syndrome), increasing the risk of VTA during periods of stress or elevated heart rates (Figure 1) [19]. Conversely, mutations that enhance I_Ks activity may shorten the APD, creating a predisposition to arrhythmia. Short QT syndrome and AF are primary arrhythmic conditions associated with this phenomenon [20].

Genetic polymorphisms, particularly SNPs, can lead to variations in the amino acid sequences of proteins or alter genetic pathways, thereby influencing an individual's susceptibility to certain diseases. Such variations can modify the structure, folding, stability, activity, and interactions of proteins, which, in turn, affect cellular functions and overall physiology. These genetic variations can predispose individuals to diseases by altering the response of proteins to stressors. However, these predispositions may not manifest phenotypically as diseases, unless they are triggered by environmental factors (ischemia, increased metabolic stress, toxins, etc.) [28, 29]. In this context, our findings suggest that coronary artery disease and secondary development of ischemic cardiomyopathy may play a triggering role. While we did not find a significant difference in serum potassium levels between the two groups, we found a significant difference in KCNQ1 gene polymorphisms, which encode channels that affect intracellular potassium levels.

**Figure 2:**
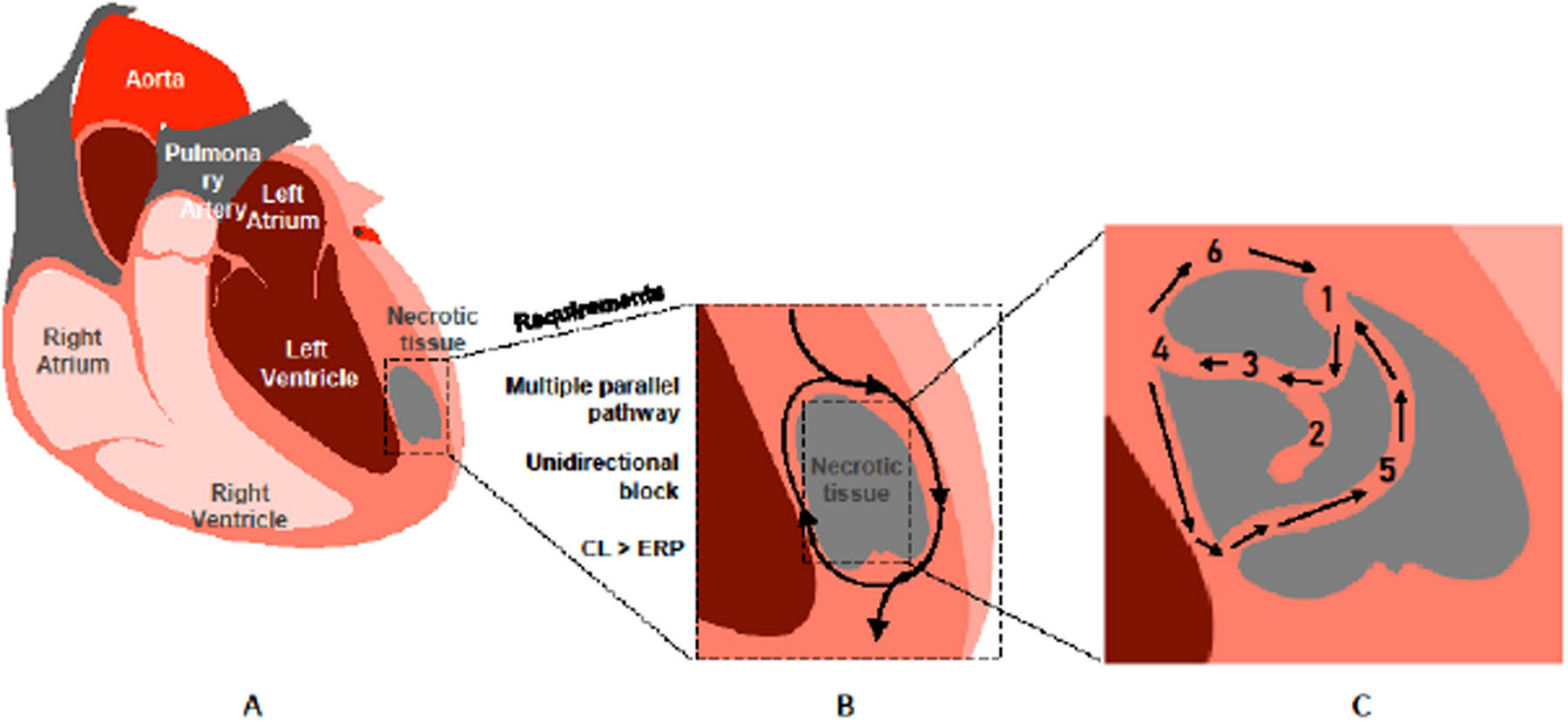
The role of necrotic tissue in cardiac conduction: Mechanisms of reentry via unidirectional block and multiple parallel pathways. Diagram illustrating the effects of necrotic tissue on cardiac conduction pathways. The presence of necrotic tissue creates unidirectional block and multiple parallel conduction pathways, facilitating reentry mechanisms. This can result in sustained arrhythmias when the conduction cycle length (CL) exceeds the effective refractory period (ERP). Abbreverations: CL, Cycle length; ERP, Effective refractory period.

Substrate structure is crucial for the development of scar‐dependent ventricular tachyarrhythmia (VTA) in patients with ischemic cardiomyopathy (Figure 2). The damaged myocardium undergoes structural and electrophysiological remodeling during the post‐infarction period. In particular, the peri‐infarct region serves as a transition zone where significant heterogeneity occurs in the electrical properties of cardiomyocytes. Changes in action potentials in cardiomyocytes in this zone, irregularities in the repolarization phase, and alterations in conduction velocity within the peri‐infarct tissue create ideal conditions for the formation of re‐entry circuits. The resulting re‐entry circuit must, on the one hand, be fast enough to maintain continuous electrical activity necessary for the persistence of tachyarrhythmia (responsiveness to supraventricular stimuli), and on the other hand, the cycle length it creates must be slow enough to allow myocytes to exit the absolute refractory period. Thus, the size and structure of scar tissue as well as factors affecting APD in peri‐infarction tissues (affecting both depolarization and repolarization phases) can modify the onset, zone, and persistence of tachyarrhythmias. Although there is typically a homogeneous conduction in the myocardial tissue, significant variations are observed in the initiation, conduction and termination rates of action potentials between different cardiomyocyte clusters in the peri‐infarction region, referred to as the “infarct border zone”. The longest segments of the APD in cardiomyocytes occur during phases 2 (plateau phase) and 3 (repolarization phase). The ion channels and electrophysiological processes that affect these phases are the determining factors for APD. The heterogeneity arising from the involvement of these phases can contribute to the formation of re‐entry circuits in two ways: 1) it can cause blockages in conduction pathways, allowing the action potential to circulate through alternative routes (usually exhibiting slowed conduction due to changes in ion channel expression and increased fibrosis, thereby increasing the likelihood of re‐entry); and 2) it can shorten the APD of the cells in the peri‐infarct region, making them re‐excitable (Figure 1). Potassium is the most important ion influencing the processes of phases 2 and 3 of the action potential, and the I_Ks channels encoded by the KCNQ1 gene play a critical role in regulating repolarization by affecting these phases. Our KCNQ1 polymorphism findings were significant in this respect. Factors such as reduced EF (negatively correlated with scar tissue size), electrolyte imbalance (potassium, magnesium, etc.), genetic polymorphisms affecting ion channels (long QT syndrome, Brugada syndrome, etc.), myocardial ischemia, age, and gender have been cited as the primary clinical risk factors associated with this mechanism [30, 31, 32, 33, 34]. However, we did not find any significant difference between the arrhythmia and control groups in these factors. We also did not find any significant difference between the two groups in terms of other risk factors reported to be associated with VTA, such as chronic kidney disease, hypertension, diabetes mellitus, etc [35, 36, 37]. The fact that we did not find any significant difference in these clinical risk factors between the arrhythmia and control groups underscores the importance of the KCNQ1 gene polymorphism.

Inflammation plays a significant role in the development of cardiovascular disease, affecting a wide spectrum from atherosclerosis to senile valve pathologies [38, 39, 40, 41, 42]. In addition to its vascular and structural effects, inflammation also plays a role in arrhythmogenesis through multifactorial mechanisms. Key factors include the formation of re‐entry circuits (resulting from fibroblast activation and accumulation of extracellular matrix protein deposits), ion channel dysfunction, increased sympathetic activation of the autonomic nervous system, and deformities in the conduction system due to inflammatory infiltration of the myocardium [43, 44, 45, 46]. Consequently, an increase in chronic metabolic inflammation has been associated with the development of arrhythmias [47, 48]. The systemic inflammatory index (SII) serves as a reliable indicator of chronic low‐grade metabolic inflammatory states [49]. We found SII to be significantly higher in the arrhythmia group than in the control group.

The KCNQ1 gene, which is critical for cardiac repolarization, has been associated with various inflammatory processes, and loss of function in this gene may lead to dysregulation in pathways featuring interleukin‐7, regenerating islet‐derived protein 3 gamma, peptidoglycan recognition protein 1, etc [50, 51]. We found a significant difference in genetic polymorphisms between the arrhythmia and control groups (*p* < 0.001). On the other hand, we did not find any significant difference between the groups in the use of statins, which have been shown to possess anti‐inflammatory effects [52].

Activation of the sympathetic nervous system facilitates the development of VTA by increasing automatism in cardiomyocytes and altering the repolarization dynamics [53]. During sympathetic activation, β‐adrenergic stimulation enhances I_Ks activity through phosphorylation by protein kinase A (PKA). This results in faster activation and slower deactivation of the potassium channels [54]. Consequently, mutations in KCNQ1 may facilitate arrhythmia development by reducing the response to parasympathetic stimulation [55]. The increase in intracellular calcium levels due to the activation of late sodium channels (I_Na) also increases sympathetic system activation [56]. In fact, cardiac drugs such as β‐adrenergic receptor blockers and ranolazine exhibit antiarrhythmic effects [44, 45]. We found no significant difference in the use of these drugs between the arrhythmia and control groups, which makes the difference in polymorphism between the groups even more significant.

It has been reported that the frequency of hospitalization in patients with ischemic cardiomyopathy is also closely related to the development of VTA due to increased risk factors, electrophysiological changes and comorbidities leading to sympathetic activation [46]. We found no significant difference between the arrhythmia and control groups in terms of these parameters, indicating that they have no statistically significant effect on the development of VTA.

Electrical stabilization of the myocardium is essential for maintaining effective circulation and optimal hemodynamic function. Conditions that lead to electrical heterogeneity in the myocardium create an arrhythmic substrate, and several ECG parameters below have been associated with this situation [53]: 1) QT_d_, which indicates heterogeneity in ventricular repolarization and is associated with the formation of substrates for re‐entrant circuits and ectopic activity; 2) frontal QRS‐T angle, which is related to the balance between ventricular depolarization and repolarization, with any disruption linked to the development of re‐entry circuits; and 3) TPEI, which reflects the transmural distribution of ventricular repolarization [57, 58, 59]. We found these parameters to be significantly higher in the arrhythmia group compared to the control group, consistent with the literature. Additionally, the correlation analyzes we conducted to assess the impact of KCNQ1 gene polymorphisms on these parameters revealed significant relationships between all genotypes of the RS2237895 polymorphism and the TT genotype of the RS2237892 polymorphism. The RS2237892 CT polymorphism, on the other hand, was solely associated with the TPEI. However, it has been shown that clinical arrhythmia does not develop in cases featuring diabetes mellitus, systemic sclerosis, compensatory autonomic regulation, age, gender differences, absence of inducible arrhythmogenic substrate, secondary responses to some medications, heart rate variability, etc., where abnormalities in these parameters are not derived from repolarization heterogeneity. Furthermore, these ECG changes cannot adequately predict arrhythmia unless they are extremely pronounced. In this context, it is crucial to assess KCNQ1 polymorphisms to verify whether ECG changes are derived from repolarization heterogeneity. Evaluating changes in KCNQ1 polymorphisms in conjunction with these ECG parameters may increase the predictability of VTA development. Thus, the risk of overdiagnosis of silent mutations that do not cause tachyarrhythmia can be avoided and the risk of arrhythmia can be assessed more accurately.

The intracellular concentration of potassium, which plays a critical role in the stabilization of the repolarization phase, is maintained by rapidly activated delayed rectifier potassium current (I_Kr) and I_Ks channels. I_Ks significantly contributes to the later phases of cardiac repolarization, particularly during the plateau phase of the action potential. This prolonged activation helps stabilize the APD. On the other hand, I_Kr, although also important, primarily affects the early repolarization phase and is more sensitive to environmental factors, e.g. extracellular potassium concentration, drug interactions, pH changes, oxidative stress, temperature and hormonal effects. Disruption of I_Ks can lead to an increase in APD, which increases the risk of arrhythmias due to prolonged refractory periods and the potential for re‐entry circuits. In cases where I_Kr is impaired due to genetic mutations, drug effects, etc., it can be partially compensated by I_Ks. The compensatory role of I_Ks underscores its indispensable function in maintaining electrical stability, continuity, and preventing arrhythmias. Understanding these dynamics is critical for developing therapeutic strategies to effectively manage ventricular arrhythmias.

Current guidelines recommend prophylaxis primarily with ICD implantation for the following three groups of patients with advanced ischemic cardiomyopathy: 1) symptomatic heart failure patients with LVEF ≤ 35% despite at least 3 months of optimal medical therapy, 2) asymptomatic patients with LVEF ≤ 30%, and 3) patients with non‐sustained ventricular tachycardia and LVEF ≤ 40%. The current guidelines’ recommendations resulted in a 5.6% reduction in risk from 19.8% to 14.2% compared with the medically managed group after 20 months of follow‐up [8, 22]. It has been reported that this risk significantly decreases by the end of the first 2 years [23].

Identifying the patient group at risk of survival despite optimal medical therapy is critical in making the decision to implant an ICD. Our findings indicate that the KCNQ1 polymorphism may be a useful parameter in distinguishing the patients at risk for VTA from among those who developed ischemic cardiomyopathy on a scar substrate. In this context, our study may help identify patients at risk for life‐threatening arrhythmias, determine the patients in need of antiarrhythmic drug support, guide device implantation decision in challenging cases due to comorbid conditions, inform decisions regarding reimplantation in patients who develop device‐related infections, facilitate monitoring during the interim process leading to reimplantation as in cases with infective endocarditis that receive home antibiotic therapy, guide make battery replacement decisions in patients without complications following ICD implantation whose devices have reached the end of their battery life and determine whether to opt for cardiac resynchronization therapy (CRT)‐pacemaker or CRT‐defibrillator devices in patients planned for CRT with low‐to‐moderate LVEF.

### 4.1 Limitations of the Study

Our study had several limitations. The primary limitation of our study was its single‐center design and relatively small sample size. Secondly, the effect of this gene polymorphism in the long‐term follow‐up remains unclear. Thirdly, although a history of known arrhythmogenic diseases was excluded, genetic analysis for existing conditions was not performed. Fourthly, the scar sizes were not compared between patients using imaging techniques such as cardiac magnetic resonance imaging (MRI) or myocardial perfusion scintigraphy. Fifthly, patients with nonischemic cardiomyopathy (NICM) were excluded due to the limited sample size in this subgroup, which restricted statistical power. Additionally, ischemic and nonischemic cardiomyopathies exhibit distinct electrophysiological and genetic mechanisms. While myocardial fibrosis and chronic ischemia are key modulators of KCNQ1 polymorphism's arrhythmogenic effects in ischemic cardiomyopathy, inflammatory processes or genetic mutations may play a more dominant role in NICM. Further studies are warranted to assess the impact of KCNQ1 polymorphism in NICM.

## 5 Conclusions

Our findings suggests that KCNQ1 polymorphism may serve an independent predictor of VTA development in patients with ischemic cardiomyopathy. The availability of such a marker is likely to facilitate clinicians’ assessment of the risk of VTA development, especially in cases where it is challenging to make the decision for ICD implantation.

## Author Contributions

Conceptualization: Uğur Özkanğur Özkanğur Özkan, Metin Budak, Kenan Yalta, Muhammet Gülnur Öztürkülnur Öztürkürdoğan, Servet Altay. Data curation: Uğur Özkanğur Özkanğur Özkan, Metin Budak, Mustafa Yildiz, Kenan Yalta, Muhammet Gülnur Öztürkülnur Öztürkürdoğan, Servet Altay. Formal analysis: Uğur Özkanğur Özkanğur Özkan, Metin Budak, Mustafa Yildiz, Kenan Yalta, Muhammet Gülnur Öztürkülnur Öztürkürdoğan, Servet Altay Altay, Gülnur Öztürkülnur Öztürkülnur Öztürk. Investigation: Uğur Özkanğur Özkanğur Özkan, Metin Budak, Kenan Yalta, Muhammet Gülnur Öztürkülnur Öztürkürdoğan, Servet Altay. Methodology: Uğur Özkanğur Özkanğur Özkan, Kenan Yalta, Muhammet Gülnur Öztürkülnur Öztürkürdoğan, Servet Altay, Gülnur Öztürkülnur Öztürkökay Taylan. Project administration: Uğur Özkanğur Özkanğur Özkan, Metin Budak, Kenan Yalta, Muhammet Gülnur Öztürkülnur Öztürkürdoğan, Servet Altay. Resources: Uğur Özkanğur Özkanğur Özkan, Metin Budak, Kenan Yalta, Muhammet Gülnur Öztürkülnur Öztürkürdoğan, Servet Altay. Supervision: Uğur Özkanğur Özkanğur Özkan, Metin Budak, Kenan Yalta, Muhammet Gülnur Öztürkülnur Öztürkürdoğan, Servet Altay Altay. Writing – original draft: Uğur Özkanğur Özkanğur Özkan, Metin Budak, Kenan Yalta, Muhammet Gülnur Öztürkülnur Öztürkürdoğan, Servet Altay, Gülnur Öztürkülnur Öztürkülnur Öztürk, Gülnur Öztürkülnur Öztürkökay Taylan.

## Ethics Statement

The study protocol was approved by the Trakya University Medical Faculty Ethics Committee (TUTF‐BAEK 2022/105).

## Conflicts of Interest

The authors declare no conflicts of interest.

### Code Availability

The authors have nothing to report.

## Data Availability

The data that support the findings of this study are available from the corresponding author upon reasonable request.
